# Supporting those experiencing food insecurity: A scoping review of the role of a dietitian

**DOI:** 10.1111/jhn.13407

**Published:** 2024-12-15

**Authors:** Aashna Kundra, Hiba Batool, Sally G. Moore, Avril Aslett‐Bentley, Sharon Noonan‐Gunning, Isabel Rice, Jo Smith, Luise V. Marino

**Affiliations:** ^1^ School of Food Science and Nutrition University of Leeds Leeds UK; ^2^ Freelance Consultant Dietitian/Nutritionist West Yorkshire UK; ^3^ Department of Sociology and Criminology City, University of London London UK; ^4^ Sustain: The alliance for better food and farming London UK; ^5^ Tees Esk and Wear Valleys NHS Foundation Trust Teeside University Darlington UK; ^6^ Research and Development South West Yorkshire Partnership Foundation Trust, Fieldhead Hospital Wakefield UK

**Keywords:** dietitians, food insecurity, policy, professional practice, training

## Abstract

**Background:**

Increasing levels of food insecurity in the United Kingdom (UK) suggest an imperative to consider the role of dietitians in supporting people who may have insufficient access to safe and nutritious food.

**Objective:**

To explore the available evidence on the (i) role of a dietitian, (ii) impact of support and (iii) training needs of dietitians to support those with inadequate access to food.

**Methods and Design:**

Scoping review methodology was used to identify qualitative, quantitative and grey literature on the role of dietitians in supporting those with/or at risk of food insecurity. The Preferred Reporting Items for Systematic reviews and Meta‐Analyses extension for Scoping Reviews (PRISMA‐ScR) was used to report the evidence reviewed for this study. Methods included multiple literature searches, charting of data extracted, and content analysis. The data of interest included the country of study, study methodology, the population of interest, the role of a dietitian, the measures, tools or guidance used and a summary of key findings.

**Results:**

In total, 466 studies were identified, following the removal of duplicate records, 243 records were screened for inclusion; the full text of 95 articles was reviewed for eligibility, and 19 were included in the review. Articles were summarised descriptively using tables and synthesised to identify emerging themes. Overarching themes of dietitians' role included, (i) identification and screening of food insecurity, (ii) facilitating community interventions and (iii) policy development.

**Conclusions:**

Dietitians hold a range of roles to support people at risk of or experiencing food insecurity. However, there are considerable gaps in current training programmes, and a paucity of evidence describing the impact dietitians have on improving nutrition outcomes for those individuals at risk of or experiencing food insecurity.

## Highlights

- Increasing levels of food insecurity in the United Kingdom (UK) make it important to consider the role of dietitians in supporting people who may have insufficient access to safe and nutritious food.
- While limited, the available literature demonstrates that dietitians play a range of roles in supporting people at risk of, or experiencing, food insecurity including identification and screening, facilitating interventions and policy development.
- Gaps exist in current training and evaluation of the impact dietitians have on improving nutrition outcomes for individuals at risk of or experiencing food insecurity.

## INTRODUCTION

The Rome Declaration on World Food Security and the World Food Summit Plan of Action |Food Agriculture Organisation (FAO) defines food security as being ‘when all people, at all times, have physical and economic access to sufficient safe and nutritious food that meets their dietary needs and food preferences for an active and healthy life’.^1^ So, food insecurity is ultimately underpinned by structural factors. The FAO Food Insecurity Experience Scale (FIES) describes food insecurity from mild (uncertainty around the ability to obtain food) to severe (no food for a day or more).^2^ Food insecurity, once considered only to be a problem in low‐ and middle‐income countries, is now reported to affect 8%–20% of households within rich countries.^3^ Children living in poverty in Organisation for Economic Co‐operation and Development (OECD) countries is increasing, with a reported prevalence in Canada 9.5%, the United Kingdom (UK) 12.7% and 20.4% in the United States America (USA), respectively.^4^ In 2013, an estimated 60 million people approximately 7.2% of the population in high‐income countries made use of a food bank.^5^ However, this trend appears to be increasing year on year, with UK data reporting that in 2008/9 around 26,000 people accessed a food bank, which had increased to approximately 2.99 million by 2022/23.^6^ The Food Foundation Food Insecurity Tracker (UK) (Survey 13; 2023) reported 9.3 million adults (17.7%) in all households experienced moderate or severe food insecurity in the preceding month.^7^

Data from the Food Foundation (UK) suggests children are adversely affected by food insecurity, with 21.6% of households reporting children directly experiencing food insecurity during the past month affecting 3.7 million children, compared with 11.6% in January 2022. However, these estimates are likely to underreport the true scale of the problem as there continue to be variations in tools and terminology used to measure and report the prevalence of food insecurity.^8^ Of households experiencing food insecurity,^8^ 38.6% are in employment suggesting remuneration from work is not always sufficient to prevent families from experiencing food insecurity,^9^ and many were healthcare workers.^9^, ^10^ A lack of nutritious and safe food is associated with an increased risk of obesity,^11^ poor dietary quality,^12^ inadequate nutrient intake,^13^ poorer mental health,^14^, ^15^ or control of physical conditions such as food allergy^16^, ^17^ and coeliac disease.^18^ In addition, children who are food insecure are more likely to report poorer health status (i.e., stunting)^19^ with more hospitalisations, increased risk of micronutrient deficiencies as well as having poor health indicators compared with children living in food secure environments.^20^

Dietitians are trained to interpret nutritional science to improve the health and wellbeing of individuals, treating mental and physical conditions through education, therapeutic diets including medical nutrition^21^, ^22^, ^23^ and dietary manipulation such as food fortification or food exclusions, which may have a significant financial impact on households budgets.^24^ They also have a critical role in public health nutrition (PHN), as part of the community‐level infrastructure that brings people together reducing isolation, supporting food insecure people and maintaining mental and physical wellbeing across the life course. An example is the dietetic helper model, of community nutrition assistants that was developed in 2000s.^25^ However, the capacity and capabilities of dietitians and public health nutritionists are argued to be reduced due to disinvestment in public health with austerity policies that were initiated in 2010.^26^ This scoping review contributes to a wider discussion on the role of dietitians in PHN. It focuses on the individual level and argues that dietitians need sufficient training, to be able to incorporate compassionate food security screening into daily clinical practice across the lifecourse^27^ and be able to identify affordable, accessible, appropriate and healthier foods as part of a nutrition care plan.^28^ The aim of this scoping review was to identify the available evidence from high‐income countries on the (i) role of dietitians in supporting households experiencing (or at risk of) food insecurity, (ii) the impact this support may have on improving mental health (including physical and nutritional health) and well‐being outcomes and (iii) training needs for dietitians with regards to food insecurity.

## METHODS

### Preparing to scope the literature protocol development

A scoping review was conducted to identify the key concepts within this area of research.^29^ The scoping study design was chosen because it offered a framework to identify and synthesise a broad range of evidence. The scoping review methodology provided an opportunity to focus on the role of dietitians in supporting those experiencing or at risk of food insecurity and help identify gaps in the literature and future research priorities.^30^ The Preferred Reporting Items for Systematic reviews and Meta‐Analyses extension for Scoping Reviews (PRISMA‐ScR) was used to develop and report the evidence reviewed for this study.^31^

### Identifying the research questions

- What is the role of dietitians in supporting households experiencing or at risk of food insecurity?
- What is the impact of dietitians on the outcomes of individuals experiencing or at risk of food insecurity?
- What are the current gaps in knowledge and training for dietitians in the UK as part of undergraduate and postgraduate training?

Using the PRISMA checklist^29^, ^31^ an a priori scoping review protocol was developed prior by A. K., H. B. further developed this, and included (1) the research question, (2) eligibility criteria of the studies be to included, (3) information sources to be searched, (4) description of a full electronic search strategy, (5) data charting process with data items included and (6) critical appraisal and synthesis of the data in order to answer the question posed.

### Data sources—Stage 1

Following the finalisation of the research question and objectives a literature search was completed to identify studies in scope. An information specialist assisted with the development of a search strategy, which was completed across seven electronic databases; MEDLINE, Cochrane, EMBASE, CINAHL, SCOPUS, Web of Science and PubMed, with searches adapted for each database. Forward and backward citation searching was completed on studies exploring the role of dietitians in supporting households with food insecurity. A 15th‐year time limit was set as 2008 (global recession) until February 2024, to ensure as much contemporaneous evidence from high‐income countries as possible was included.

### Search strategy—Stage 2

A search strategy was devised with the assistance of an information specialist for PubMed using keywords from the grey literature and modified for additional electronic databases (Supporting Information S2: Table 1). Grey literature portals including governmental, higher education institutes (HEIs) nutrition, professional registration requirements and nutrition professional organisation websites and resources within high‐income countries were also searched.

**Table 1:** Development of codes, subcategories and overarching themes.

Initial coding (*n* = 80)	Subcategories (*n* = 10)	Overarching themes/categories (*n* = 3)
Explore the role of a dietitian Referral pathway Factors influencing food insecurity Identification of food insecurity Clinical responsibility Barriers to assessment Recommendations for assessment and management Practical and ethical uncertainties working with food insecure Nutrition care process Screening tool Who can screen Who should screen Possible actions High food security Marginal food security Low food security Very low food security Food insecurity informed care	Screening and assessment Nutrition care process Food insecurity informed care	Identification and screening for food insecurity
Fostering collaboration Educating public and health professionals Advocating for global security Diversity of professional understanding and experience with food insecurity Nutrition knowledge, training and understanding Education and practice Research Advocacy and public policy Policymakers Culturally appropriate foods Confidence of healthy eating More than the food model Recipe cards New food environment Dietary guidelines Healthy eating recommendations National Food and Nutrition Strategy Capacity‐building activities to equip current and future nutrition and dietetic workforce Lack of experience Clinician's knowledge of food insecurity Impacts on mental and socio‐economic wellbeing Food as a medicine Evaluation and outcome standards Health and quality of life Determinants of exposure to risk factors Multifaceted response Healthcare utlisation and cost Best practice resources Public health emergency	Education, training and policy Cultural competence Intervention impact and outcomes	Training and policy development
Personal and environmental factors Communicate health message Culinary medicine programme Low income Culinary medicine Promoting healthy eating Reducing the risk of chronic disease Cultural diversity Special Supplemental Nutrition Programme for Women, Infants and Children Federal school nutrition programmes Adaptation Food banks Food pantry Food insecurity Food choice Impact Physical, social, mental and health outcomes Immigrants and refugees Children Ante‐natal Pregnancy Maternal and child health Older adults Diabetes Obesity COVID‐19 Telemedicine Barriers and challenges Public health nutrition role Sustainable foods Negative impacts of food insecurity on health condition management Conditions influenced by diet Medication regimes influenced by food Inter‐agency working Intervention development Intervention implementation Breadth – the entire community Social change Education skills and ecology Duration – sustainability Survey	Community‐based initiatives Food banks and food pantries Social, physical and mental health conditions Healthy and sustainable food	Facilitating community interventions

### Study selection—Stage 3

Titles and abstracts were screened by two reviewers (A. K., H. B.). Duplicates were deleted, full text articles were reviewed for eligibility. S. M. acted as a third reviewer when agreement around inclusion/exclusion was not reached. Inclusion criteria included any study that used a qualitative and quantitative design, studies in English including published thesis and conference abstracts, and those involving dietitians or dietitian nutritionists in relation to food insecurity as defined by the FAO.^1^ Systematic reviews were not included, but the references of studies were hand searched for any references, which may fulfil the inclusion criteria. Exclusion criteria were publications not in the English language or those that did not relate to registered dietitian or dietitian nutritionist healthcare professionals.

### Data extraction—Stage 4

Data extraction was completed using a two‐stage process. A data extraction template (Microsoft 2010) was created and used to capture the study design, results and conclusions, followed by a content analysis.

### Collating, summarising and reporting the results—Stage 5

Data synthesis was completed using an established content analysis approach,^32^ this method was chosen as a technique for reporting common themes within data.^33^ This approach captured descriptive aspects of the study, methodology, outcomes and any key findings that were coded. A content analysis was completed by selecting, coding and creating initial codes, subcategories and overarching themes to develop into a conceptual framework.

## RESULTS

### Selection and characteristics of included articles

**Figure 1:**
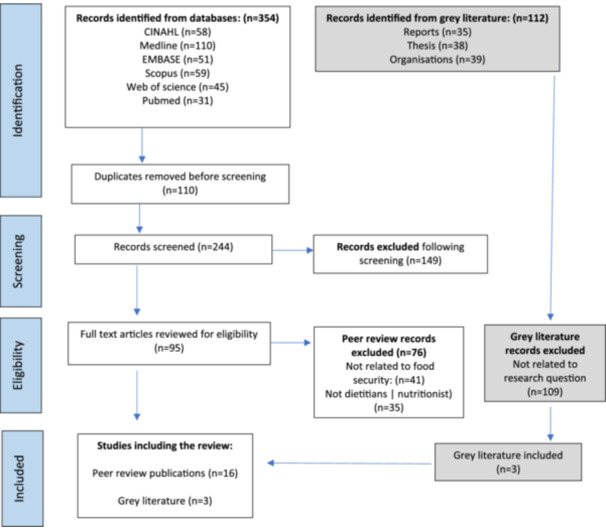
Searches through to inclusion.

A total of 466 records were identified, including information from the grey literature. Following the removal of duplicate records, abstracts and titles of 243 records were screened for inclusion. The full text of 95 articles was reviewed for eligibility, of which 19 were included (Figure 1).

#### Content analysis: Conceptual framework and overarching themes

A content analysis identified 80 codes, 10 subthemes and 3 overarching themes, which were identified (Table 1) as:

1. *Identification and screening for food insecurity;* (i) screening and assessment, (ii) nutrition care process and (iii) food insecurity informed care
2. *Training and policy development;* (i) education, training, and policy and (ii) cultural competence, and (iii) intervention impact and outcomes,
3. *Facilitating community interventions;* (i) community‐based initiatives, (ii) food banks and food pantries, (iii) social, physical and mental health conditions and (iv) healthy and sustainable food

**Figure 2:**
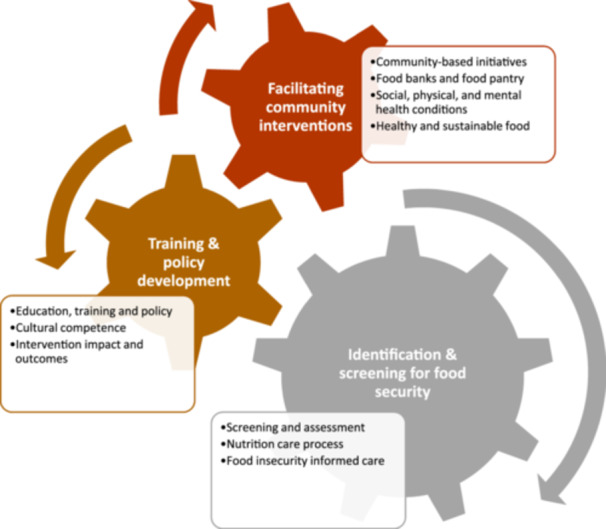
Conceptual framework of the interdependencies and the role of a dietitian in supporting those who are food insecure.

These were used to develop a conceptual framework of the interdependencies and the role of a dietitian in supporting those who are food insecure (Figure 2).

#### Study characteristics

Nineteen studies were included in the scoping review that examined the role of dietitians in supporting those with food insecurity including; curriculum framework,^34^ Delphi process,^35^ position(practice) guideline,^36^, ^37^, ^38^ observational,^39^, ^40^, ^41^, ^42^ review,^43^, ^44^, ^45^, ^46^ survey^47^, ^48^ and thesis.^49^, ^50^, ^51^ Articles reported clinical, community and household areas of practice in Australia (*n* = 2),^36^, ^52^ Canada (*n* = 2),^35^, ^43^ USA (*n* = 10),^38^, ^39^, ^40^, ^44^, ^45^, ^46^, ^47^, ^48^, ^50^, ^53^ UK (*n* = 4),^34^, ^41^, ^49^, ^51^ and Europe (*n* = 1)^37^ (Table 2).

**Table 2:** Characteristics of studies describing the role of dietitians in supporting those with food insecurity.

References	Title	Country	Methodology	Population of interest	Role of a dietitian	Measure/Tools/Guidance	Key findings
Alexandra Harper^49^	A Nutrition Education and Cooking Intervention in a UK Foodbank	United Kingdom	Observational (Thesis)	Adults	Education and cooking intervention – food bank	Questionnaire	Healthy behaviour change noticed in adults, and the nutrition knowledge and confidence of participants increased.
Barbour et al.^36^	Dietitians Australia position statement on healthy and sustainable diets	Australia	Position Statement	Adults	Dietitians have a key role to play in contributing to food system transformation, in particular by facilitating a population‐wide shift to healthy and sustainable diets, ensuring they are affordable.		Dietitians Australia recommends: (i) the development of a National Food and Nutrition Strategy, which recognised indigenous knowledge of food systems, (ii) the integration of sustainability principles in Australia's dietary guidelines, (iii) the reorientation of our food environment to prioritise access to healthy and sustainable foods and (iv) investment in capacity building activities to equip the current and future nutrition and dietetics workforce including aspects related to food security.
British Dietetic Association^34^	Curriculum Framework 2020	United Kingdom	Curriculum framework	Adults	Food provision, including production, procurement and delivery, and food security and sustainability	Curriculum Framework	Pre‐registered dietitians are expected to have a broad knowledge and understanding of food security and sustainability.
Burton‐Obanla et al.^53^	Oncology registered dietitian nutritionists' knowledge, attitudes and practices related to food insecurity among cancer survivors: A qualitative study	USA	Observational	Oncology patients	Screening for food insecurity	Telephone interview (49 min) with (n = 41) dietitians	No tools or communication techniques were used to assess clients' food insecurity status, supporting the urgent need to develop education and training opportunities among oncology registered dietitian nutritionists (RDNs)
Carlson et al.^54^	How Can Dietitians Leverage Change for Sustainable Food Systems in Canada?	Canada	Delphi process	Adults	The development of future food systems in 15 thematic areas of the social and ecological systems. Barriers are described according to how they undermine sustainability and the role of a dietitian within them.	58 dietitians	High‐leverage action areas included: (i) facilitating knowledge development within the profession and public (including food security), (ii) influencing organisational policy to support sustainable food systems and (iii) influencing public policy. Approaches to such action included: (i) facilitating cross‐sectoral collaboration and (ii) applying reflexive approaches
Dickson et al.^52^	Antenatal healthcare providers' knowledge, attitudes and practices regarding food insecurity in pregnancy: A qualitative investigation based at a specialist antenatal hospital in Melbourne, Australia	Australia	Observational	Adults	Opportunities to identify and effectively manage women who are food insecure during pregnancy to support optimal maternal and foetal health	Semi‐structured qualitative interviews	Current assessment and management of food insecurity in an antenatal setting is suboptimal. Antenatal healthcare providers (including dietitians) had limited knowledge and awareness of food insecurity during pregnancy. This group of healthcare professionals lacked experience in managing food insecurity and time pressures meant there was insufficient time available to support and deliver care to food‐insecure women.
Douglas et al.^41^	'Health professionals’ experiences and perspectives on food insecurity and long‐term conditions: A qualitative investigation	UK	Observational	Adults	Experiences and views of HCPs (including dietitians) in North East Scotland, focusing on support for people with long‐term conditions whom they believed were affected by food insecurity.	Semi‐structured qualitative interviews	HCPs (including dietitians) had (a) *diverse levels of understanding and experience of food insecurity*, they identified a range of (b) *negative impacts of food insecurity on condition management*, especially for diet‐dependent conditions or medication regimes, and for mental health. Even for those health professionals more familiar with food insecurity, there were various (c) *practical and ethical uncertainties about identifying and working with food insecure patients* (it could be difficult to judge, for example, whether and how to raise the issue with people, to tailor dietary advice to reflect food insecurity and to engage with other agencies working to address food insecurity). HCPs working with food‐insecure patients have learning and support needs that warrant further investigation. There are numerous debates around HCPs' responsibilities, and interventions to guide and support HCPs, including tools that might be used to screen for food insecurity, and reflect the diverse lived needs and values of people who experience food insecurity.
Downer et al.^45^	Food is medicine: actions to integrate food and nutrition into healthcare	USA	Review	Adults	Interventions involving tailored meals and grocery delivery linked to nutrient patterns among food‐insecure clients	Evaluation of different interventions	The need for dietitians and other clinicians to obtain adequate education and training to be able to support vulnerable groups was prioritised.
European Federation of the Associations of Dietitians^37^	The role of European public health dietitians	Europe	Position statement	Adults (Individual and group settings)	Nutrition education, training, advice and practice, and innovative research related to all aspects of food insecurity	Data collected from US households (Surveys)	Public health dietitians are involved in the delivery and development of policies related to nutritional well‐being and food justice across the life course. Enhancing health equity by advocating for reorienting food and health policies.
Holben, Marshall^38^	Position of the Academy of Nutrition and Dietetics: Food insecurity in the United States	USA	Position statement	Adults	Suggesting programmes to reduce food insecurity	Analysis of the collection of studies	Several ways to help – programmes to promote healthy eating, elaborated on the practices used by dietitians (counselling, policy development, education, workshops)
Handu et al.^47^	Preparing future registered dietitian nutritionists for working with populations with food insecurity: A new food insecurity/food banking supervised practice concentration piloted with dietetic interns	USA	Cross‐sectional anonymous survey	Adults (Farmer population)	Raise awareness of major issues in global food security, examine agriculture practices and innovations and highlight the importance of different actors like RDNs in the food systems to combat food insecurity	RDNs and farmers were interviewed	It is critical for dietitians to understand agriculture and the food systems, so they may help their patients gain access to sustainable healthy foods.
Homenko et al.^39^	Food Insecurity and Food Choices in Rural Older Adults with Diabetes Receiving Nutrition Education via Telemedicine	USA	Observational	Older adults	Awareness of diabetes nutrition education via telemedicine	Telephone survey (n = 74)	97% of adults purchased fresh produce and took the dietitians' advice while purchasing food and noticed changes in overall health.
Kelley et al.^48^	Repeated cross‐sectional surveys of registered dietitian nutritionists demonstrate rapid practice changes to address food insecurity during the coronavirus 19 disease 2019 pandemic	USA	Cross‐sectional anonymous survey	Adults	Dietitians had around 10 years' experience in addressing food insecurity and were most commonly involved in the Special Supplementation Nutrition Programme from Women, Infants and Children, federal school nutrition programmes or food banks.	Wave 1: n = 454 Wave 2: n = 331	In response to the COVID‐19 pandemic, dietitians were quick to adapt programmes to ensure clients' and staff's safety, while continuing to provide essential food security services. Dietitians identified the need for ongoing nutrition programme policy advocacy and access to best practice resources during public health emergencies.
Lycett D, 2020^51^	A Feasibility Study of a Brief Intervention for Food Insecurity in Dietetic Practice: British Dietetic Association (BDA)	United Kingdom	Mixed methods(Thesis)	Food‐insecure clients	Screening and recommendations	Questionnaire/survey, telephonic conversations for follow‐up	5 dietitians agreed to screen n = 50 at‐risk individuals as part of routine clinical appointments. They were provided training prior to the use of the tool. 28% of the participants were identified as food insecure and all of them accepted the BDA leaflet recommendation.
McWhorter et al.^40^	Barriers and Facilitators of Implementing a Clinic‐Integrated Food Prescription Plus Culinary Medicine Programme in a Low‐Income Food Insecure Population: A Qualitative Study	USA	Observational	Low‐income populations with chronic disease	Training and education of RDN's (Registered dietitian nutritionists)	Set up focus groups (patients with diabetes and RDN employees)	Lack of culturally inclusive dietary recommendations and a desire to develop skills in preparing tasty and healthy food were reported by patients. RDNs emphasised having better training and education in cultural humility, culinary nutrition skills and behavioural change theory.
Ramsahoi, Sonny, Monk, 2022^43^	Exploring Barriers to Food Security Among Immigrants: A Critical Role for Public Health Nutrition.	Canada	Review	Immigrants	In accessing, obtaining and consuming their cultural foods	Observational study design followed by qualitative data collection.	Dietitians play a key role by serving as a conduit for new immigrants to access community resources – personalised health coaching, 1‐2‐1 appointments and motivational interviewing skills
Vogliano et al.^55^	Plentiful, Nutrient‐Dense Food for the World: A Guide for Registered Dietitian Nutritionists.	USA	Position statement	Adults	Spreading information to those enroled with food banks and providing efficient food and recipe preparation techniques	Surveys	Not many people understood the complicated science‐based terms used, hence this should be converted into practical information for better understanding to increase nutrition education. Suggested the need for change in food banks, and the delivery of education.
Wetherill et al.^44^	Food Insecurity and the Nutrition Care Process: Practical Applications for Dietetics Practitioners	USA	Review	Older adults/Families	Application of the Nutrition care process and identifying different steps to screening food insecurity and developing community programmes beneficial for food‐insecure clients.	A 10‐item U.S. Adult Food Security Survey Module (Survey based)	Signposting action items for dietetics practitioners/nutrition professionals to support the delivery of food insecurity‐informed client care.
Willians et al.^50^	Registered Dietitian Nutritionists' Perceptions Helping Mothers Gain Sustainable Access to Healthy Foods.	USA	Observational (Thesis)	Mothers	Registered Dietitian Nutritionists assess their knowledge and awareness of assistance programmes	Cross‐section online survey involving 533 RDNs	This survey is framed by the community readiness model. Most RDNs had no knowledge of nutrition assistance programmes designed to gain access to healthy food.

##### Identification and screening for food insecurity

(a) Screening and assessment

There are several considerations for food insecurity screening for dietitians, including the most appropriate tool to be used (the determination of which was outside of the scope of this review). Within their position statement, the Academy of Nutrition and Dietetics endorses a two‐question screening tool, with a 97% sensitivity and 74% specificity for household food insecurity.^38^ Answering ‘often true’ or ‘sometimes true’ to either question below suggests household food insecurity.^27^, ^56^

1. ‘Within the past 12 months, we worried whether our food would run out before we got money to buy more (available responses: often true, sometimes true, never true)
2. Within the past 12 months, the food we bought just didn't last and we didn't have money to buy more (available responses: often true, sometimes true, never true)’

A feasibility study explored the use of the two‐question screening tool^27^, ^56^ in a dietetic‐led clinic, as part of a social prescribing initiative exploring making every contact count.^57^ Five dietitians were trained to screen (*n* = 50) at‐risk individuals. More than 25% of the participants were classified as food insecure. Of these individuals 92% accepted information on the BDA Food insecurity leaflet and 42% preferred foodbank vouchers. However, no outcomes relating to the impact on nutritional status or health‐related quality of life of this intervention were described.^51^

(b) Nutrition care process

Dietetic professional bodies (Australia,^36^ Europe,^37^ USA^38^) have developed position statements around food insecurity, supporting food security screening within the Nutrition Care and Process model providing dietitians with the opportunity to signpost individuals to support and advice to minimise the risk of or consequence of being food insecure, as well as providing information relating to research and advocacy efforts.

(c) Food insecurity informed care

The British Dietetic Association (BDA) curriculum framework identified that preregistered dietitians should have a knowledge and understanding of ‘food provision, including production, procurement and delivery and food security and sustainability’.^34^ The Academy of Nutrition and Dietetics has developed practice guidelines incorporating food insecurity screening into the nutrition care process. Practice recommendations for the provision of food‐insecurity informed care include (i) ensuring food insecurity screening systems are in place when working with at‐risk populations, (ii) making use of screening data to support critical appraisal during the assessment process, (iii) being aware of the impact of household food insecurity on individual health and the capacity to implement medical and nutrition treatment plans, (iv) maintaining an up to date list of supportive voluntary care organisations, (v) exploring collaborations with local food partners to develop medically tailored food distribution and (vi) modifying goals as required when evaluating individual progress and evaluation outcomes in response to the nutrition intervention.^44^

##### Training and policy development

(i) *Education, training and policy*
  A training Food Insecurity/Food Banking Supervised Practice Concentration programme was developed, the purpose of which was to better prepare for preregistration dietitians and dietitians working with populations with a high prevalence of food insecurity.^47^ Within the BDA curriculum framework model, knowledge and understanding of food security and sustainability is required.^34^ The American Dietetic Association provides a number of statements under Education and Practice in the Position of the Academy of Nutrition and Dietetics‐Food Security in the United States,^44^ these include (i) incorporating food‐security‐related concepts and experiential learning into a preregistration curriculum for dietitians, (ii) promote and encourage students to participate in food security or food bank practice experience, (iii) conduct screening using the two‐question validated tool,^27^, ^56^ (iv) communicate food‐insecurity‐related information to professionals, legislators, policymakers and community leaders as an advocate for change and improving outcomes,^44^ and developing global standards of practice.^46^
  A cross‐sectional survey of dietitians working in North Carolina (USA), considered aspects relating to the scope of practice and knowledge and were asked to rate the importance of helping mothers who were food insecure. All survey respondents felt helping mothers was important and within their scope of practice; however, 19% of dietitians were unaware of programmes supporting mothers to access nutrition assistance and support. Of the 81% who were aware they could name an average of 3 ± 1.7 state and national support programmes,^50^ suggesting more training is required. Similar findings have been reported amongst immigrants,^35^, ^43^ low‐income groups,^40^ antenatal care for pregnant women^52^ and cancer sufferers.^53^
(ii) *Cultural competence*
  As part of this project a survey of food bank managers (*n* = 100) was conducted, the majority of whom (60%) reported working with dietitians and dietetic interns. Results from the survey reported that although food bank managers appreciated the work of dietitians/dietetic interns, they felt many lacked the necessary skills for working with this vulnerable population group, which included their inability to translate science‐based information into practical information, lack of knowledge on food ingredients and how to prepare food in environments with limited facilities.^47^
  These findings are echoed by others with regards to dietitians requiring cultural competence around foods commonly eaten as part of cultural food traditions.^35^, ^40^, ^42^, ^43^, ^50^ A qualitative study explored healthcare professionals' (including dietitians) experiences and perspectives on food insecurity in individuals with long‐term conditions indicating several learning and training needs. A thematic analysis of qualitative findings reported (i) a diversity of understanding and experience of food experience, (ii) negative impacts of food insecurity on condition management (including medication adherence) and (iii) significant practice and ethical uncertainty about identifying and responding to food insecure patients.^41^
(iii) *Intervention impact and outcomes*
  Few studies reported food security intervention and its impact on social, mental and physical health outcomes. Homenko et al.^39^ explored the relationship between food insecurity and the ability of older rural individuals with diabetes to buy and make suitable meals when receiving remote nutrition counselling via telephone from dietetic diabetes educators. Individuals who were mildly insecure (23%) were significantly more likely to have a higher body mass index (35.5 ± 7.1 kg/m^2^ vs. 30.5 ± 6.0 kg/m^2^, *p* = 0.01), lower household income (*p* = 0.03), and more likely to consider the cost of food ingredients (*p* = 0.03) compared with adults who were food secure. Both groups reported similar adherence to dietitians' advice and had similar glycemic control. In another study, five dietitians were trained to screen (*n* = 50) at‐risk individuals using a validated two‐question food insecurity screening tool.^27^ Of these individuals, 92% accepted information (British Dietetic Association [BDA] food insecurity leaflet) and 42% preferred foodbank vouchers. More than 25% were classified as food insecure. However, no outcomes relating to the impact on nutritional status or health‐related quality of life of this intervention were described.^51^ Other interventions describe improved client knowledge around physical activity recommendations (55%), the Eatwell guide plate (40%) and confidence around meal planning (*p* < 0.0001).^49^

##### Facilitating community interventions

(i) *Community‐based initiatives*
  Dietetic‐led projects supporting food bank users have been completed, an example of which was implemented in a food bank in Coventry (UK), where food bank users were provided two sessions involving information and practical skills session around cooking a healthy meal. Forty‐two foodbank clients completed the intervention, at the end of which there was a significant improvement in the client's knowledge of The Eatwell Guide (40%) (*p* < 0.001). Fruit and vegetable consumption was low (on average 2 portions per day), with cost being cited as a barrier. At the end of the intervention, clients felt significantly more confident in planning a meal (*p* < 0.001), suggesting dietitians may have an impact on improving nutrition well‐being outcomes for individuals with food insecurity.^49^
(ii) *Food banks and food pantries*
  Dietitians working in public health can rapidly adapt to address food insecurity.^48^ However, other groups working in clinical settings may not be quite so agile, more so in those working with individuals with multimorbidity. Social prescribing of healthy food prescriptions may also serve to improve the overall health status with the help of medically tailored groceries and producing general health prescriptions to balance nutrient intakes could benefit clients with food insecurity. However, for this to be achieved, dietitians need to receive adequate training and education for them to implement community‐based programmes.^45^ Dietitians can also play a role in establishing nutritional adequacy of food banks and food pantry parcels.^58^
(iii) *Social, physical and mental health conditions*
  Dietitians working with cancer survivors,^53^ low‐income mothers^50^ or individuals with low‐income and chronic diseases,^40^ did not routinely assess individual risk of food insecurity or use a validated screening tool suggesting a need to develop education and training tools with regard to cultural humility and practical culinary nutrition skills to help to improve acceptability and adherence to nutrition care plans of individuals with poor food security across the physical health spectrum. These findings are echoed within other studies with regard to cultural competence around foods commonly eaten as part of cultural food traditions.^35^, ^40^, ^42^, ^43^, ^50^
(iv) *Healthy and sustainable food*
  Dietitians should also be involved in policy development, a Delphi process identified diet food systems, health and nutrition promotion with the inclusion of plant‐based foods (sustainable) for individual and planetary health should be promoted to protect food security at all levels of the food system, encouraging nutrient‐rich foods along with plant‐based diets,^35^, ^36^, ^39^ along with influencing legislation and government policy,^45^ social marketing, reforming school environments to help in nutrition education, and research.^37^

## DISCUSSION

The results of this scoping review suggest dietitians have an important role to play in supporting individuals and households with/or at risk of food insecurity. From the available evidence dietitians appear to be involved in three overarching areas, including *(i) Identification and screening for food insecurity, (ii) Training and policy development and (iii) Facilitating community interventions*. Dietitians play important roles within communities that are vulnerable to food insecurity and have the ability to respond quickly to healthcare emergencies.^48^

There are several considerations for food insecurity screening for dietitians, including the most appropriate tool to be used, and outside of the scope of this review. Within their position statement, the Academy of Nutrition and Dietetics endorses a two‐question screening tool, with a 97% sensitivity and 74% specificity for household food insecurity.^38^ Routine screening for food insecurity is crucial given the cost of living continues to escalate.^59^ Since April 2023 in the UK food and fuel prices are reported to have risen by more than 19.1% and 40.5%, respectively.^60^ As a result many in employment find they have insufficient money for food and fuel placing households at risk of fuel poverty and food insecurity, increasing the risk of poor mental and physical health outcomes, particularly for children.^59^ The BDA has launched a poverty and food security campaign,^28^ including a position statement addressing the (i) scale of food poverty in the UK, (ii) cause of food poverty and (iii) the impact of food poverty^61^ and ultimately the end of the need for food banks. However, gaps exist with regard to training needs analysis and support for dietitians across the life course with regard to the identification and support for households with or at risk of insufficient access to food, and the resources needed to enable dietitians in this work. As such there is an urgent imperative to implement considerations for the ability of households to access sufficient food by including food security screening with the BDA Nutrition Care Process model,^23^ using a validated food insecurity screening tool such as ‘Hunger Vital Sign’.^27^, ^56^ The Academy of Nutrition and Dietetics has adopted food security screening into the Nutrition Care Process Model,^38^, ^44^ and there are isolated examples of the use of validated screening tools in practice in the UK, such as a nutrition awareness tool for children,^62^ but it is not known how many dietitians routinely use screening tools as part of their clinical practice and whether they are embedded within Nutrition and Hydration Guidelines^63^ or department standards within health and social care settings.

Dietitians working in food banks and food pantries have been shown to support individuals with a variety of healthcare needs to gain access to healthy food,^47^ improve nutritional outcomes^39^ and knowledge with regard to healthy diets.^49^, ^50^, ^51^ Dietitians working in the area of food sustainability can support change through advocacy, research, education, training, prevention policymaking and community activities by developing strategic alliances and collaborations with other stakeholders and organisations to ensure that sufficient healthy food to meet nutrition requirements is available for all within a sustainable food system.^8^, ^36^, ^38^, ^64^ Within this context, dietitians should also be able to understand factors influencing food choices in the context of nutrition and dietetic practice,^65^ and support healthy and sustainable diets for individual and planetary health.^36^

While some dietitians feel it is within their scope of practice to identify and discuss aspects relating to food security,^50^ some studies identified training and education needs across the professional spectrum from preregistration through the experienced practitioners,^38^, ^50^, ^53^ especially with regards to cultural competence around foods commonly eaten as part of cultural food traditions^35^, ^40^, ^42^, ^43^, ^50^ and ethical uncertainty about identifying and responding to food insecure patients.^41^ Our findings suggest that although dietitians recognise the importance of supporting individuals with food insecurity, there may be insufficient training from preregistration and across the professional spectrum.^44^, ^45^, ^47^, ^50^, ^53^ As such providing future and current dietetic practitioners with knowledge and skills to support people experiencing food insecurity could improve the food security status of individuals and households,^38^ and for dietitians within the UK, there is a moral and social imperative for urgent upskilling with regard to providing food security informed care. However, as few studies reported on outcomes, this may indicate a need to support dietitians to more effectively measure impact and outcomes, by providing training. Understanding the interdependencies of food insecurity and poor dietary quality, dietitians could positively impact reducing future disease burden through well‐designed community‐based interventions that aim to improve quality of life and reduce chronic diseases of lifestyle.

## LIMITATIONS

There are several limitations to this work, relating to the paucity of research regarding the role of a dietitian in supporting those affected by fluctuating access to sufficient food. The quality of the evidence is varied and was not formally assessed in line with the scoping review methodology. This scoping review identified several gaps in the research, including the impact of dietetic support on important health (physical and nutritional) and well‐being outcomes, and sustained access to food. There were also gaps in knowledge, training and education needs across the spectrum of the profession from preregistration to specialist dietitian, which requires further exploration. Finally, the role and influence dietitians may have in terms of lobbying for local, regional and national guidelines was also not well described.

## CONCLUSION

Dietitians hold a range of roles to support people experiencing food insecurity. However, there are considerable gaps in current training programmes, and a paucity of evidence describing the impact dietitians have on improving nutrition outcomes for those individuals who are experiencing food insecurity.

## AUTHOR CONTRIBUTIONS

Sally G. Moore and Isabel Rice conceptualised the study and research question with Luise V. Marino, Jo Smith, Sharon Noonan‐Gunning and Avril Aslett‐Bentley, following which Aashna Kundra and Hiba Batool undertook the literature searches, article identification data extraction and synthesis guided by Sally G. Moore. Aashna Kundra, Hiba Batool, Sally G. Moore and Luise V. Marino drafted the manuscript, and all authors provided revisions and approved the manuscript.

## CONFLICT OF INTEREST STATEMENT

LVM has received an honorarium for providing educational talks for Abbott Laboratories, Danone and Nestle.

### PEER REVIEW

The peer review history for this article is available at https://www.webofscience.com/api/gateway/wos/peer-review/10.1111/jhn.13407.

## Supporting information

Supporting information.

Supporting information.

## Data Availability

The data that support the findings of this study are available from the corresponding author upon reasonable request.
