# Supplementary material for: Supporting those experiencing food insecurity: A scoping review of the role of a dietitian
Source: J Hum Nutr Diet. 2024 Dec 15;38(1):e13407. doi: 10.1111/jhn.13407 (PMC11647070; doi:10.1111/jhn.13407)
Supplement: Supplementary file 2 — Supporting information. [file JHN-38-0-s001.docx]

**Supplementary File 1: Table 1 Search strategy**

| **Database** | **Search Strategy** | **Results** |
| --- | --- | --- |
| Medline | Search field: All fields; Search term: (food insecurity OR food security OR food insecure OR access to healthy foods OR food access OR food hunger OR food poverty OR food bank* OR nutrient intake*) AND (nutritional professional* OR dietitian* OR nutritionist* OR dietician*); No limits used. | N=189 |
